# Genome-wide identification and role of *MKK* and *MPK* gene families in clubroot resistance of *Brassica rapa*

**DOI:** 10.1371/journal.pone.0191015

**Published:** 2018-02-14

**Authors:** Yinglan Piao, Kaining Jin, Ying He, Jiaxiu Liu, Shuang Liu, Xiaonan Li, Zhongyun Piao

**Affiliations:** College of Horticulture, Shenyang Agricultural University, Shenyang, China; Chungnam National University, REPUBLIC OF KOREA

## Abstract

Mitogen-activated protein kinase (MAPK or MPK) cascades play key roles in responses to various biotic stresses, as well as in plant growth and development. However, the responses of MPK and MPK kinase (MKK) in Chinese cabbage *(Brassica rapa* ssp. *pekinensis)* to *Plasmodiophora brassicae*, a causal agent of clubroot disease in *Brassica* crops, are still not clear. In the present study, a total of 11 *B*. *rapa MKK* (*BraMKK*) and 30 *BraMPK* genes were identified and unevenly distributed in 6 and 10 chromosomes, respectively. The synteny analysis indicated that these genes experienced whole-genome triplication and segmental and tandem duplication during or after the divergence of *B*. *rapa*, accompanied by the loss of three MKK and two MPK orthologs of *Arabidopsis*. The *BraMKK* and *BraMPK* genes were classified into four groups with similar intron/exon structures and conserved motifs in each group. A quantitative PCR analysis showed that the majority of *BraMKK* and *BraMPK* genes were natively expressed in roots, hypocotyls, and leaves, whereas 5 *BraMKK* and 16 *BraMPK* genes up-regulated in the roots upon *P*. *brassicae* infection. Additionally, these 5 *BraMKK* and 16 *BraMPK* genes exhibited a significantly different expression pattern between a pair of clubroot-resistant/susceptible near-isogenic lines (NILs). Furthermore, the possible modules of MKK-MPK involved in *B*. *rapa*-*P*. *brassicae* interaction are also discussed. The present study will provide functional clues for further characterization of the MAPK cascades in *B*. *rapa*.

## Introduction

Biotic stresses are the main threat factors for plant survival and crop production. To defend against pathogen attack, plants have evolved a variety of defense mechanisms at the molecular and cellular levels [1]. One of the earliest defense responses is the activation of mitogen-activated protein kinase (MAPK) cascades when the invading pathogens are sensed by the pattern recognition receptors (PRRs) and resistant (R) proteins [2]. The MAPK cascades are highly conserved in all eukaryotes, defining the key functional signaling modules that usually consist of three gene families—MAPKs or MPKs, MPK kinases (MKKs or MEKs), and MKK kinases (MAPKKKs or MEKKs) [3]. The components of MAPK cascades are sequentially activated via phosphorylation mediated by their upstream kinases [4]. MEKK, the first component of the MAPK cascades, phosphorylates the threonine and serine residues in the conserved S/T-X_3–5_-S/T domain of MKKs. Finally, the cascades are terminated through the MKK-mediated phosphorylation of tyrosine and threonine residues in the TEY/TDY motifs of MPKs [5]. Subsequently, the activated MPKs phosphorylate the transcription factors and enzymes that participate in the synthesis of antimicrobial metabolites, reprogramming of host genes, and synthesis of phytohormones, such as jasmonic acid (JA) and salicylic acid (SA), triggered by necrotrophic and biotrophic pathogens, respectively [6]. Thus, the stress signals are amplified and transferred into appropriate intracellular responses through MAPK cascades [7].

The genome sequencing projects enable the identification of all members of the MAPK cascades in plant species, including *Arabidopsis*, rice, pepper, and other crops, allowing for intensive functional studies. A total of 20 MPKs, 10 MKKs, and 80 MEKKs were identified in *Arabidopsis* [3, 8], whereas 17 MPKs, 8 MKKs, and 74 MEKKs were found in rice [9–11]. However, the expansion of *MPK* gene families is found in Chinese cabbage, *Brassica rapa* [12]. Generally, the number of MKKs is much less than that of their upstream and downstream kinases in the cascades. Plant MPKs are phylogenetically classified into four subfamilies, in which groups A, B, and C of MPK contain the conserved TEY domain and group D contains more MPKs sharing the TDY domain. All members of four MKK subfamilies contain the S/T-X_3–5_-S/T domain. Although the MAPK cascades are evolutionally conserved, sequence diversities exist between the members of both MPK and MKK gene families [8]. Recently, the emergence of a new version of *B*. *rapa* genome sequences allowed for the systematic characterization of gene diversity and further MAPK cascades in *B*. *rapa* [12].

Previous studies have shown that the transcripts of MPKs, MKKs, and MEKKs are specifically modified by biotic and abiotic stimuli. For instance, the *MPK3*, *MPK4*, and *MPK6* genes can be activated by pathogen elicitors and regulate innate immune responses in *Arabidopsis* [13–15]. The *AtMKK3* gene can enhance the tolerance against the bacteria *Pseudomonas syringae* by regulating a pathogenesis-related (PR) gene [14]. *OsMPK5* can be induced by various pathogens and abiotic stresses in rice, and has been found to positively regulate drought, cold, and salt tolerance, but negatively regulate the PR gene expression and resistance to fungal (*Magnaporthe grisea*) and bacterial (*Burkholderia glumae*) pathogens [16]. Several MAPK cascades involved in plant-pathogen interactions and abiotic stress responses have been functionally characterized. For example, MEKK1-MKK4/5-MPK3/6 and MEKK1-MKK1/2-MPK4 promote the expression of *Arabidopsis* resistance genes against *Pseudomonas syringae*; MEKK1-MKK1-MPK4 can transmit drought and wounding signals [15,17]. MAPKKKα/ε-MEK2-SIPK/WIPK/NTF4 and NPK-MEK1-NTF6 induce a hypersensitive response in tobacco [18]; MAPKKKα-MKK2-MPK1/MPK2/MPK3 is involved in disease resistance response in tomato [19]; MKK4-MPK1 is involved in wounding in rice [20]. Furthermore, a single MAPK cascade might be involved in multiple environmental stress responses. For example, the activation of CRLK1-MEKK1/MKK2-MPK4/MPK6 cascade has been reported in salt and cold stress responses in *Arabidopsis* [21].

*Brassica rapa* includes a variety of valuable vegetable crops, such as Chinese cabbage, pak choi, turnip, etc., as well as oilseed crops, such as turnip rape and yellow sarson. These and other cruciferous species are seriously challenged by the clubroot disease caused by *Plasmodiophora brassicae*, a soil-borne, obligate biotrophic parasite. Unlike other plant pathogens, *P*. *brassicae* belongs to Rhizaria, a supergroup of protists, and has a distinct intracellular lifestyle with two infection stages, including the primary infection of root hair and the secondary infection of root cortex [22, 23]. After successful infection, *P*. *brassicae* leads to the abnormal hyperplasia of roots and subsequent yield losses. Breeding resistant cultivars is considered to be the most efficient approach to control the clubroot disease of *Brassica* species. So far, several clubroot resistance (CR) genes have been identified and used for resistance breeding [24, 25], and two *CR* genes with toll interleukin-1 receptor (TIR)–nucleotide binding (NB)–Leu-rich repeat (LRR) domain have been cloned in *B*. *rapa* [26, 27]. However, the resistance mechanism remains to be clarified. The functional characterization of *P*. *brassicae*-triggered MAPK cascades is a feasible approach for understanding the CR mechanism since MAPK cascades usually act as important signaling modules in plant innate immune systems. However, the mechanism by which the component of MAPK cascades is transcriptionally modified by *P*. *brassicae* and the roles of these cascades in *B*. *rapa*-*P*. *brassicae* interaction remain to be identified.

In this study, we aimed to identify the MKK and MPK family genes of *B*. *rapa* to characterize their gene structures and evolution and to analyze gene expression pattern in different tissues of *B*. *rapa*. The MKK and MPK genes involved in the interaction between *B*. *rapa* and *P*. *brassicae* were also identified based on their expression profiles in a pair of Chinese cabbage near-isogenic lines (NILs), carrying the clubroot-resistant or -susceptible genes at the *CRb* locus. The results obtained in this study have paved the way for a functional study of the *P*. *brassicae*-triggered MAPK cascades in *B*. *rapa*.

## Materials and methods

### Identification of *MKK* and *MPK* family genes in *B*. *rapa*

To identify the *MKK* and *MPK* genes in *B*. *rapa*, 10 *Arabidopsis thaliana* MKK and 20 MPK protein sequences were used as the seed sequences to search against *B*. *rapa* genome database BRAD (http://brassicadb.org version 1.5 and version 2.0) by using the basic local alignment search tool (BLASTP) program with a cut-off expect value of 100. The genomic and protein sequences of *Arabidopsis* were downloaded from TAIR (http://www.arabidopsis.org). The predicted *B*. *rapa MKK* and *MPK* genes were nominated as *BraMKK1* to *BraMKK9* and *BraMPK1* to *BraMPK20*, respectively, as suggested in *Arabidopsis* nomenclature (S1 Table). To ensure that no more related MPK and MKK genes existed in *B*. *rapa*, the identified BraMPKs and BraMKKs were used as query sequences to search against BRAD (http://brassicadb.org) using the BLASTP program. Similarly, *B*. *napus* and *B*. *oleracea MPK* and *MKK* genes were identified using the BLAST-like alignment tool (BLAT) CNS-Genoscope database (http://www.genoscope.cns.fr/brassicanapus/) and *B*. *oleracea* database (http://brassicadb.org/brad/blastPage.php), and named as in *B*. *rapa*.

The databases NCBI-CDD (https://www.ncbi.nlm.nih.gov/Structure/cdd/wrpsb.cgi), SMART (http://smart.embl-heidelberg.de/), and Pfam (http://pfam.sanger.ac.uk/) were used to find the conserved domains of BraMKK and BraMPK. The molecular weights (kDa) and isoelectric points (pI) of BraMKKs and BraMPKs were calculated by the Compute pI/Mw tool of ExPASy (http://web.expasy.org/compute_pi/). Their subcellular locations were predicted using TargetP and PProwler in WoLF PSORT (http://www.genscript.com/wolf-psort.html) and BaCelLo (http://gpcr.biocomp.unibo.it/bacello/pred.htm).

### Gene structure, conserved domain, motif analysis of BraMKK and BraMPK

The exon/intron structures of *BraMKK* and *BraMPK* genes were analyzed by their coding sequences with corresponding genomic sequences and obtained figures using the Gene Structure Display Server (GSDS; http://gsds.cbi.pku.edu.cn/) and adorned by Adobe Photoshop CC (http://www.photoshop.com). The conserved motif structures of *BraMKK* and *BraMPK* genes were identified by using the online analysis tool MEME (http://meme-suite.org/tools/meme).

### Sequence alignment and phylogenetic analysis of Brassica MPK and MKK

The multiple alignments of all BraMKK, BnaMKK, BolMKK, BraMPK, BnaMPK, and BolMPK protein sequences were fulfilled using Clustal Omega (http://www.ebi.ac.uk/Tools/msa/clustalo/) and tinted by using BoxShade (http://www.ch.embnet.org/software/BOX_form.html). The phylogenetic trees were constructed using the neighbor-joining (NJ) statistical method in MEGA7 program for *Brassica* MKKs and MPKs. In the phylogenetic tree, the confidence level of each branch was estimated as 0~100 by using 1000 bootstrap replications [28].

### Chromosomal location of *BraMKK* and *BraMPK* genes

The positional information of all identified *MPK* and *MKK* genes in *B*. *rapa*, *B*. *napus*, *B*. *oleracea* and *A*. *thaliana* was obtained from the above-mentioned corresponding databases and mapped to their chromosomal locations using Circos (http://circos.ca/). In addition, the *BraMKK* and *BraMPK* genes were mapped onto the *B*. *rapa* chromosomes using the Mapchart (Version 2.1) software. The evolutionary relationships between *BraMKKs*, *BnaMKK*, and *BolMKK* and between *BraMPKs*, *BnaMPKs*, and *BolMPKs* were analyzed based on their chromosomal locations and compared with *Arabidopsis* MKKs and MPKs.

### Plant materials and treatments with *P*. *brassicae*

In our previous study, a pair of Chinese cabbage near-isogenic lines (NILs) carrying either the clubroot-resistant allele of *CRbCRb* (CR BJN3-2) or the clubroot-susceptible allele of *crbcrb* (CS BJN3-2) were developed [29]. The CR BJN3-2 and CS BJN3-2 plants were sown in 50-hole plugs containing a sterile substrate and cultivated in a culture room maintained at 24°C under a photoperiod of 16 h light/8 h dark. Previous studies showed that *P*. *brassicae* can penetrate the root hairs at 12 hours after infection (hai) [30]. Therefore, the 21-day-old seedlings were inoculated with *P*. *brassicae* (10^7^ spores/mL), and then the second true leaves, hypocotyls, and roots were collected at 0, 10, 12, 13, 14, 16, and 20 hai from both CR BJN3-2 and CS BJN3-2 plants. Each treatment was biologically replicated thrice, with each replicate containing five plants. Plants treated with sterile water were sampled at 0 hai and used as controls for the real-time (RT) PCR analysis. All samples were frozen immediately in liquid nitrogen and stored at −80°C until use.

### RNA isolation and semi-quantitative and quantitative RT-PCR analyses

Total RNA was extracted from 100 mg of each sample using the TRIZOL reagent (Invitrogen, USA), according to the manufacturer’s instructions. The concentration of RNA samples was determined by using 1% agarose gel electrophoresis and NanoDrop Spectrophotometer (Thermo Scientific, USA). The first strand of cDNA was synthesized using the FastQuant RT Kit (with gDNase) (TIANGEN Biotech, China). The primers used for RT-PCR were designed using the software Primer Premier 5.0 (S4 Table). For *BraMKK1-1* and *BraMKK1-2*, the same primer pair was picked up because of their highly similar sequences.

To select the possible *P*. *brassicae*-triggered *BraMKK* and *BraMPK* genes, the cDNA pools from the leaves, hypocotyls, and roots of the CS BJN3-2 plants, were made with different samples collected at different time points to perform a semi-quantitative RT-PCR. The expression patterns at different tissues were analyzed. Each reaction was carried out in a volume of 20 μL with 2 μL diluted cDNA, 2 μL specific primers (1.0 μM), 10 μL 2X Premix Taq (TaKaRa Taq Version 2.0 plus dye, Japan), and 6 μL RNase-free ddH_2_O. The following PCR program was used: initial denaturation at 95°C for 3 min, followed by 35 cycles of denaturation at 95°C for 30 s, annealing at 60°C for 30 s, and extension at 72°C for 30 s, and a final extension at 72°C for 5 min. The *actin* and *18S rRNA* genes of *B*. *rapa* were used as the reference genes.

The *BraMKK* and *BraMPK* genes, which were differentially expressed in the roots of *P*. *brassicae*-treated CS BJN3-2 plants, were used for further analysis by a quantitative RT-PCR. Each reaction was carried out in three independent biological and technical replicates, and contained 2 μL of diluted cDNA, 2 μL of specific primers (1.0 μM), 10 μL of 2X SuperReal PreMix Plus (with SYBR Green I) (TIANGEN Biotech, China), and 6 μL of RNase-free ddH_2_O in a total volume of 20 μL. The qRT-PCR reactions were conducted in a Bio-Rad CFX96 real-time PCR system (Bio-Rad, USA) using the following reaction conditions: initial denaturation at 95°C for 4 min, followed by 40 cycles of denaturation at 95°C for 10 s, annealing at 63°C for 20 s, and extension at 73°C for 25 s. Following amplification, the melting curves were obtained by increasing the temperatures from 55 to 95°C at the intervals of 0.5°C every 10 s to confirm the specificity of PCR amplification. The *actin* and *18S rRNA* genes of *B*. *rapa* were used as reference genes and internal controls for the comparative C_T_ method of quantitation. The relative expression levels of *BraMPK* and *BraMKK* genes were calculated by the comparative cycle threshold (ΔΔCT) method.

## Results

### Identification and annotation of *MKK* and *MPK* genes in *B*. *rapa*

A total of 11 *MKK* genes were identified in *B*. *rapa* by applying 10 *A*. *thaliana* MKK protein sequences as the reference sequences to search against the local Brassica Database (BRAD version 2.0). And then, the identified 11 *MKK* genes in *B*. *rapa* were used as query sequences to search against BRAD (http://brassicadb.org) using the BLASTP program. The two analysis steps suggested an identical result. The orthologous genes of *AtMKK7*, -*8*, and -*10* were not detectable in *B*. *rapa*. In addition, only 30 *B*. *rapa* MPK genes homologous to 20 AtMPKs were identified, not 32 as reported in a previous study [12]. The *MPK11* and *MPK14* genes were missing in *B*. *rapa*.

The physicochemical parameters were also predicted for each *BraMKK* and *BraMPK* gene. The 11 identified BraMKK proteins ranged from 308 (BraMKK9) to 544 (BraMKK2) amino acids in length. Their relative molecular weights ranged from 34.29 (BraMKK9) to 60.84 (BraMKK2) kDa and their pIs ranged from 5.48 (BraMKK3) to 9.33 (BraMKK4-1). The physicochemical parameters of all proteins are listed in Table 1. The BraMKK proteins showed variable subcellular locations, with four of them located in the cytoplasm, two in the nucleus, two in the mitochondria, and one each in the plasma membrane, peroxisomes, and chloroplasts. Two sets of MKK paralogs (*BraMKK1-1/1-2* and *BraMKK4-1/4-2*) were distributed in different subcellular locations and two sets (BraMKK5-1/5-2 and BraMKK6-1/6-2) in the same subcellular location. Among the 30 *BraMPK* genes, 11 are located in the nucleus, 10 in cytoskeleton, five in peroxisomes, two in cytoplasm and one each in chloroplasts and vacuoles. Five sets of MPK paralogs (BraMPK6-1/6-2, -7-1/7-2, -12-1/12-2, -17-1/17-2/17-3, and -19-1/19-2) were located in the same subcellular location and five sets (BraMPK8-1/8-2, -10-1/10-2/10-3, -16-1/16-2, -18-1/18-2, and -20-1/20-2) in different locations (S1 Table). These results indicate the functional diversity of *MKK* and *MPK* genes in *B*. *rapa*.

**Table 1:** *MKK* genes in *B*. *rapa* genome and their sequence characteristics and physicochemical properties.

Name	Gene ID	*A*.*thaliana* ID	Group	Gene Length (bp)	Protein Length (aa)	Mol. Wt. (KD)	PI	Intron Number	Subcellular location	
									PProwler	TargetP
BrMKK1-1	BraA03005649	AT4G26070	A	2083	310	34.54361	6.19	7	Other	cyto
BrMKK1-2	BraA03005651	AT4G26070	A	2216	355	39.36718	6.71	7	Other	nucl
BrMKK2	BraA01000849	AT4G29810	A	3641	544	60.84307	7.61	13	Other	cyto
BrMKK3	BraA08002528	AT5G40440	B	2265	518	57.56042	5.48	8	Other	nucl
BrMKK4-1	BraA08000717	AT1G51660	C	1066	355	39.29151	9.33	0	CTP	plas
BrMKK4-2	BraA05000831	AT1G51660	C	1012	337	37.24358	9.17	0	Other	pero
BrMKK5-1	BraA01002775	AT3G21220	C	994	331	36.65592	8.12	0	CTP	mito
BrMKK5-2	BraA03004232	AT3G21220	C	991	330	36.58185	8.99	0	MTP	mito
BrMKK6-1	BraA03001347	AT5G56580	A	1874	358	39.92869	5.95	7	MTP	cyto
BrMKK6-2	BraA10001670	AT5G56580	A	1893	356	39.74451	5.94	7	MTP	cyto
BrMKK9	BraA07002948	AT1G73500	D	925	308	34.28858	6.99	0	MTP	chlo

### Phylogenetic and domain analysis of *Brassica* MPKs and MKKs

To reveal the evolutionary relationships of *Brassica* MKKs and MPKs, these proteins were also searched in *B*. *oleracea* (2n = 18, C genome) and *B*. *napus* (2n = 38, AC genome). The above-mentioned nomenclature of MKKs and MPKs was followed. In total, 13 BolMKKs and 25 BolMPKs and 21 BnaMKKs and 54 BnaMPKs were identified from *B*. *oleracea* and *B*. *napus*, respectively. The number of BnaMKKs and BnaMPKs retrieved in this study was more than that from a previous study [28].

**Fig 1:**
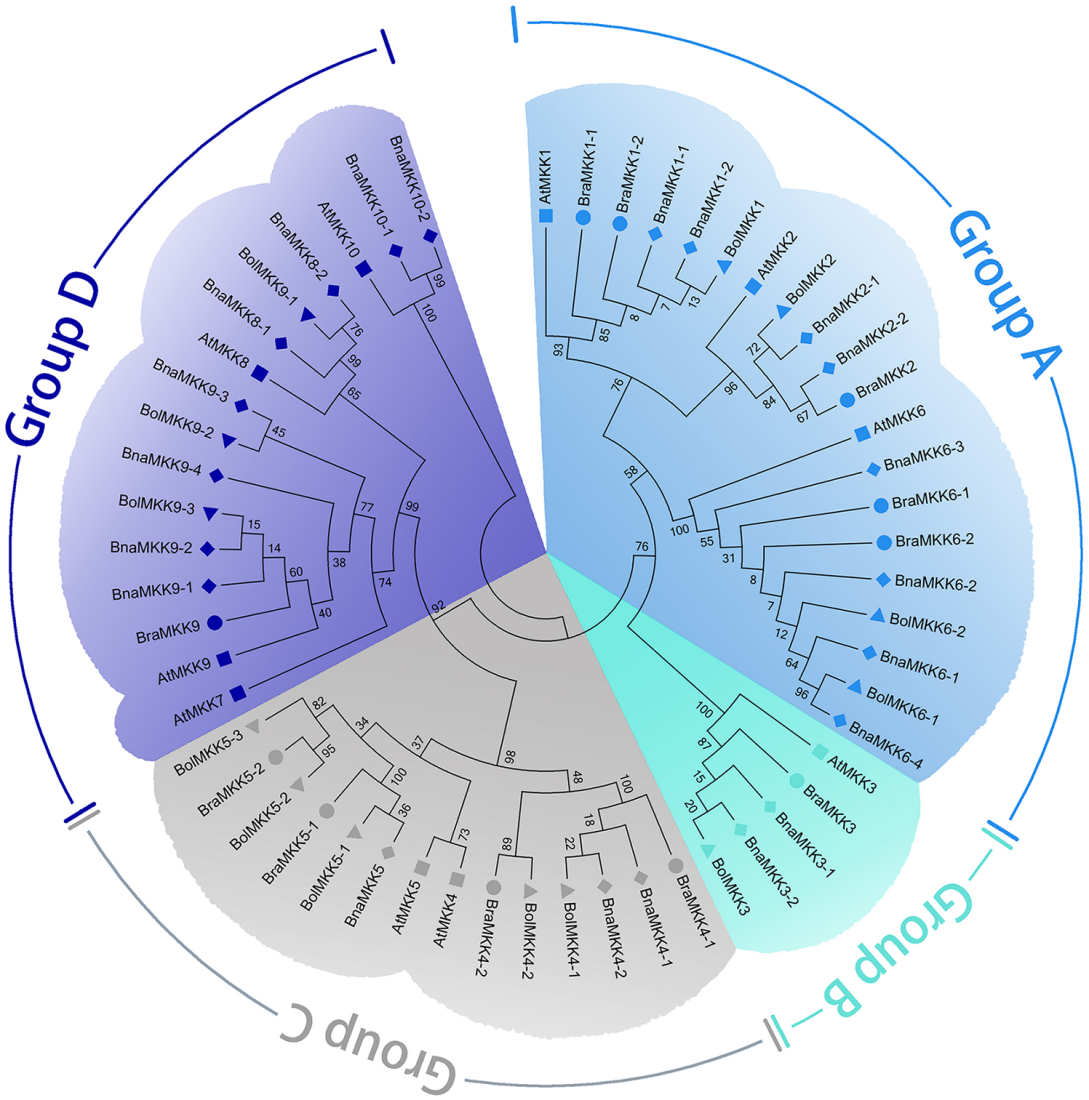
Phylogenetic tree of *MKK* genes from *B*. *rapa*, *B*. *napus*, *B*. *oleracea* and *A*. *thaliana*. The unrooted trees were constructed by using ClustalW in MEGA6 via the neighbor-joining (NJ) methods. Different species are indicated in different labels. *A*. *thaliana*: ■; *B*.*rapa*: ●; *B*.*napus*: ◆; *B*.*oleracea*: ▲. MKKs of *B*. *rapa*, *B*. *napus*, *B*. *olearacea* and *A*. *thaliana* were divided into four groups (A-D) highlighted by different colors, respectively.

**Fig 2:**
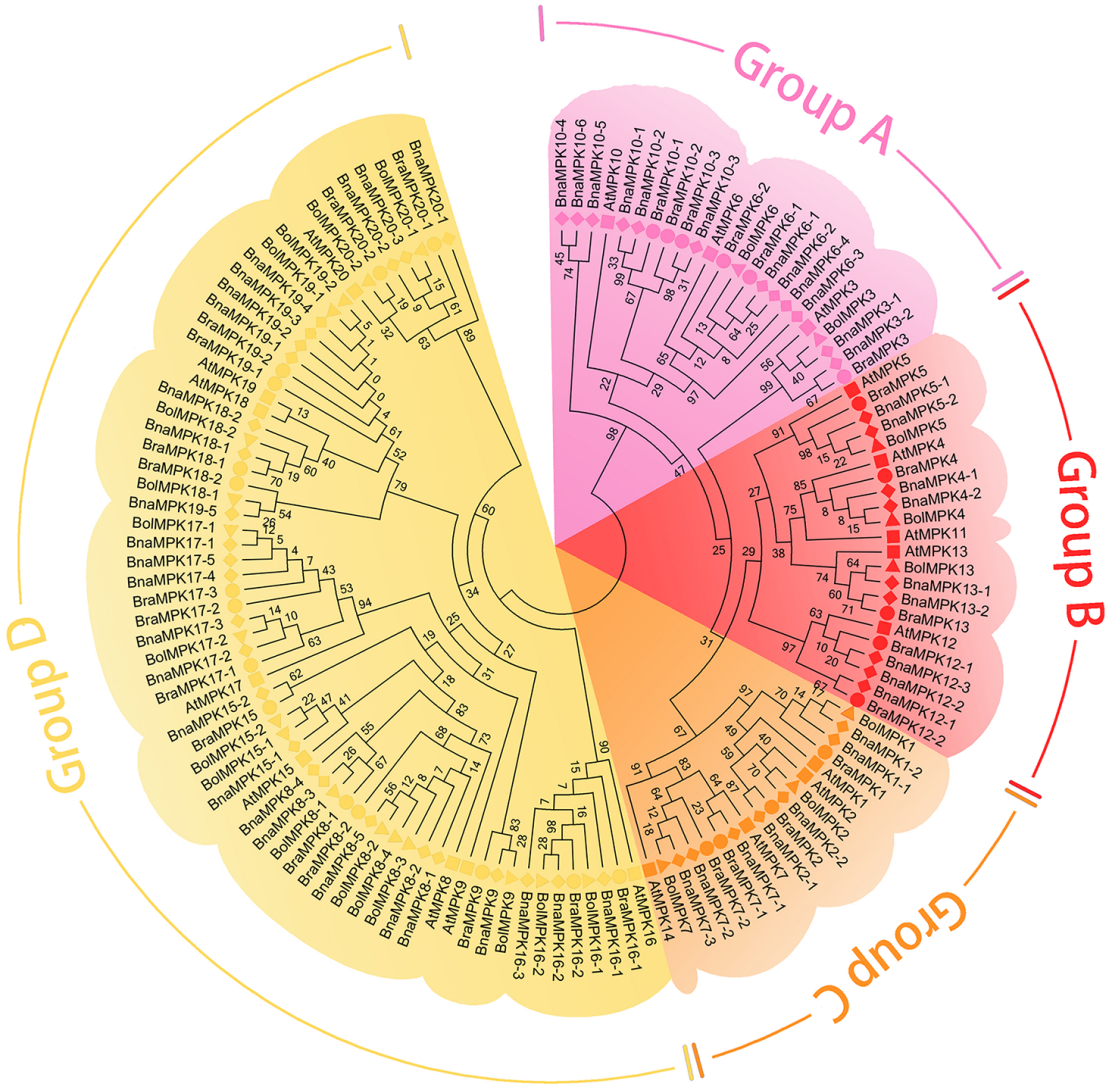
Phylogenetic tree of *MPK* genes from *B*. *rapa*, *B*. *napus*, *B*. *oleracea* and *A*. *thaliana*. The unrooted trees were constructed by using ClustalW in MEGA6 via the neighbor-joining (NJ) methods. Different species are indicated in different labels. *A*. *thaliana*: ■; *B*.*rapa*: ●; *B*.*napus*: ◆; *B*.*oleracea*: ▲. MKKs of *B*. *rapa*, *B*. *napus*, *B*. *olearacea* and *A*. *thaliana* were divided into four groups (A-D) highlighted by different colors, respectively.

The amino acid sequences of *Brassica* MKKs or MPKs and the corresponding sequences of *Arabidopsis* MKKs or MPKs were used to construct a phylogenetic tree. In agreement with *Arabidopsis*, the *Brassica* MKKs and MPKs were classified into A, B, C, and D groups (Figs 1 and 2). Notably, the *Brassica* MKKs in group C show divergence because the branch lengths of group C are shorter than those of the other groups. The multiple alignments of amino acid sequences showed that BraMKKs contained the conserved motif S/T-X_3–5_-S/T, which is known as the phosphorylation site (S1 Fig), while BraMPKs with a highly conserved TEY motif were classified in groups A, B, and C and those with a TDY motif were classified in group D (S2 Fig).

**Fig 3:**
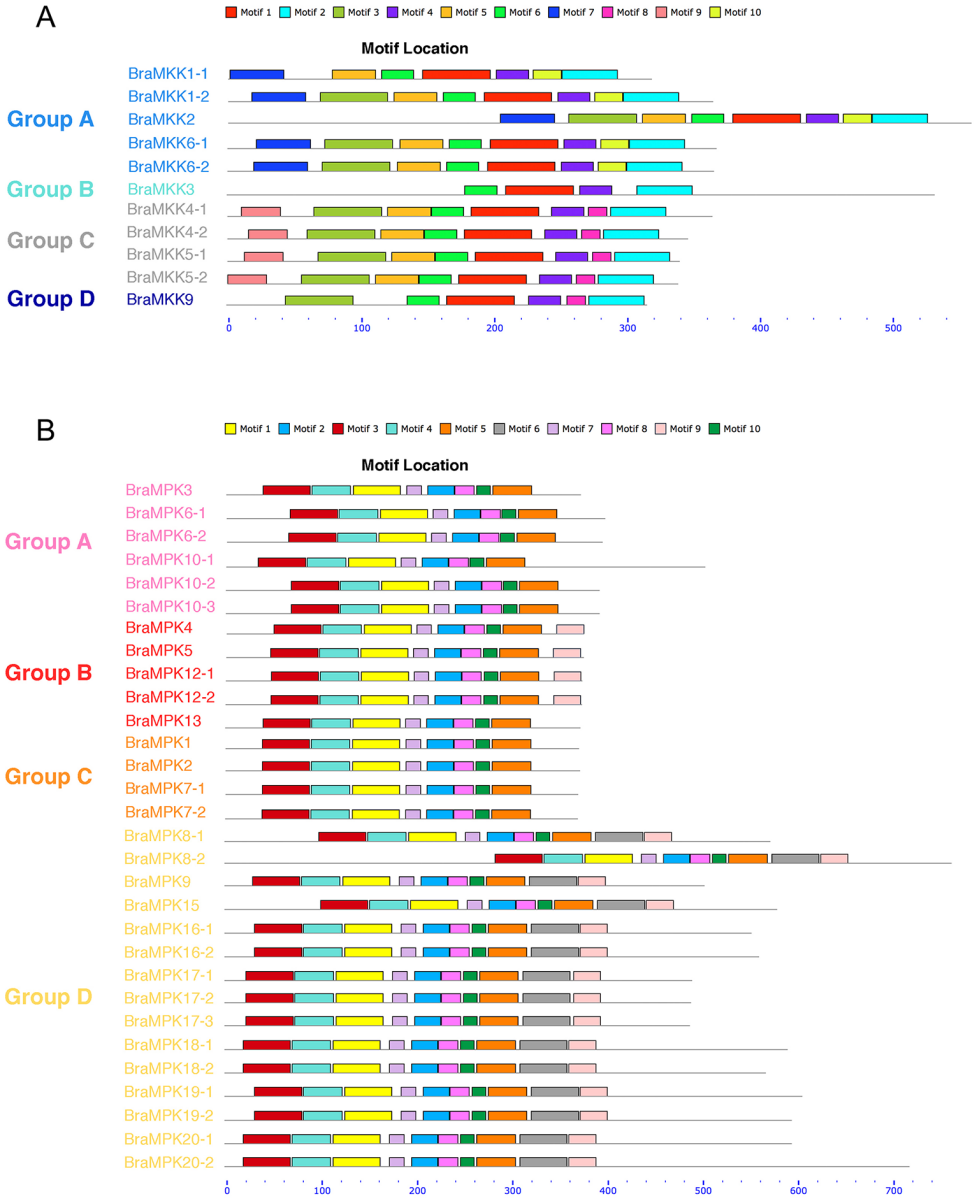
Motif analysis of *MKK* (A) and *MPK* (B) gene families in *B*. *rapa*. Protein sequences of BraMKKs and BraMPKs were used for alignment by MEME online program. BraMKKs and BraMPKs are grouped to the phylogenetic classification (A-D). Different colors of the squares represent different motifs and the grey lines indicate non-conserved sequences. The length of proteins and motifs can be estimated by scale at the bottom.

Ten conserved motif domains of BraMKKs and BraMPKs were investigated by aligning protein sequences using the MEME program. All BraMKKs were conserved in their main motifs (1, 2, 4, and 6), while motif diversity was also found between different groups. Motif 7 and 9 belonged to groups A and C, respectively. Group B is more unique; it contains only four main motifs. The paralogous pairs in group C are consistent, while the imparities in group A (BraMKK1-1/1-2 and BraMKK6-1/6-2) are due to the lack of motifs 3 and 7 in BraMKK1-1 and BraMKK6-2, respectively. Groups B and D are imperfect in motif 5 compared to groups A and C (Fig 3A and S3A Fig). BraMPKs possess 8–10 motifs. Motif 6 is specific to group D. Both motifs 6 and 9 were lost in groups A and C, but only motif 6 was lost in group B. These motifs can be used as the indicators of each group of BraMPKs (Fig 3B and S3B Fig).

### Structural analysis of *BraMKK* and *BraMPK* genes

**Fig 4:**
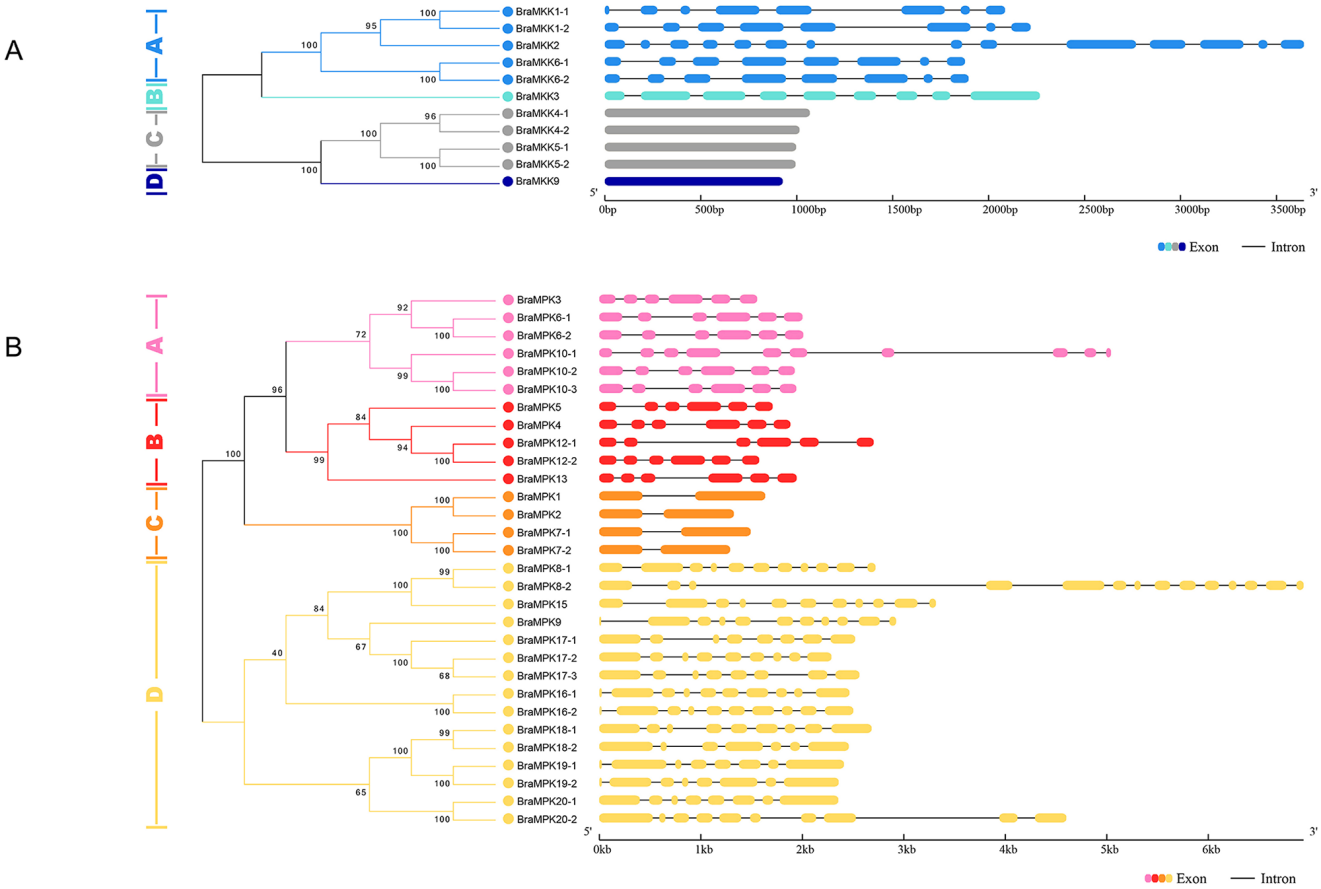
The exon/intron structure of *MKK* (A) and *MPK* (B) genes in *B*. *rapa*. Exons are represented by colorful boxes, and introns are reprensented by black lines. Introns and exons of *BraMKKs* and *BraMPKs* are grouped according to the phylogenetic classification (A-D). The exon and intron sizes can be estimated by the scale at the bottom.

To further study the gene structural evolution and analyze the structural characteristics, the coding sequences of *MKK* and *MPK* genes were aligned to the genomic sequences. The results showed that the *BraMKK* genes in groups A and B contained 7–8 introns, except for *BraMKK2* that contained 13 introns, while the genes belonging to groups C and D had no introns (Fig 4A). The protein lengths of all BraMKKs are similar, indicating that the difference in their gene structures is mainly due to the variation in their intron numbers and lengths. All paralogous gene pairs showed the same pattern of the number of exons/introns and similarity in the length of exons/introns. The intron/exon structures of most *BraMPK* genes were consistent with the previous study [12]. However, the intron numbers of the *BraMPK8-2*, -*10-1*, and -*18-2* genes showed differences in version 2.0, and were corrected to be 13, 9, and 6, respectively (Fig 4B).

### Chromosomal location and synteny analysis of *Brassica MKK* and *MPK* genes

**Fig 5:**
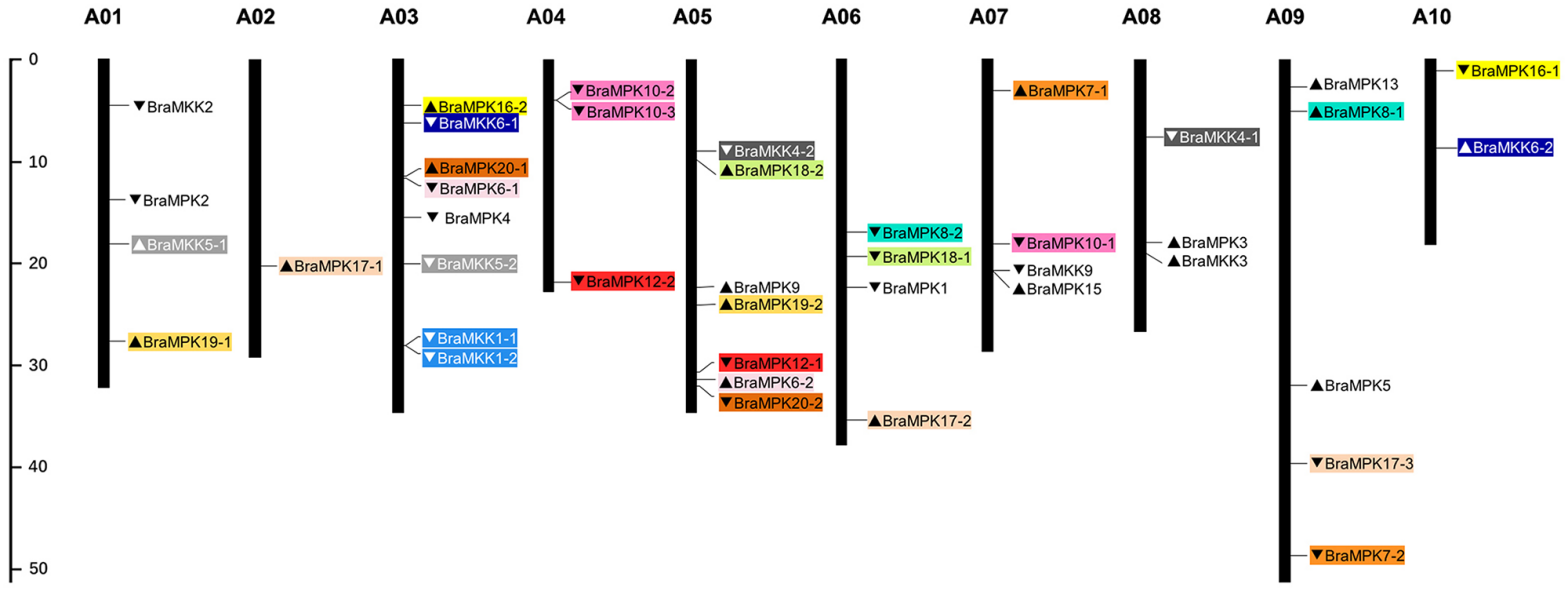
Distribution of *BraMPKs* and *BraMKKs* on *B*. *rapa* chromosomes. Paralogous genes were represented by same colors and single-copy genes were colorless. Black arrows indicated the direction of genes in corresponding chromosomes. Chromosome locations of *BraMKKs* and *BraMPKs* can be estimated by scale in the left. 11 *BraMKKs* were mapped to 8 out of 10 chromosomes. 30 *BraMPKs* were mapped to 6 out of 10 chromosomes.

Based on the physical positions of *BraMKK* and *BraMPK* genes, their chromosomal locations were determined on 10 chromosomes of *B*. *rapa* (Fig 5). The *BraMKK* genes were distributed on six chromosomes: four genes on ChrA03, two genes each on ChrA01 and ChrA08, one gene each on ChrA05, -07, and -10. The *MKK* genes located on ChrA03 showed tandem duplication, while the other paralogous pair of genes was dispersed on two chromosomes. Thirty *BraMPK* genes were distributed among 10 chromosomes: six genes on ChrA05, five genes on ChrA09, four genes each on ChrA06 and ChrA03, three genes each on ChrA04 and ChrA07, and two genes on ChrA01. Three chromosomes (ChrA02, -08, and -10) contain only one gene. Compared to the *Arabidopsis MKK* and *MPK* genes, a majority of *BraMKK* and *BraMPK* genes showed segmental duplication and only two genes (*BraMPK10-2/10-3* and *BraMKK1-1/1-2*) showed tandem duplication. These results indicated that segmental duplication played an important role in the gene evolution of *BraMKK* and *BraMPK*.

**Fig 6:**
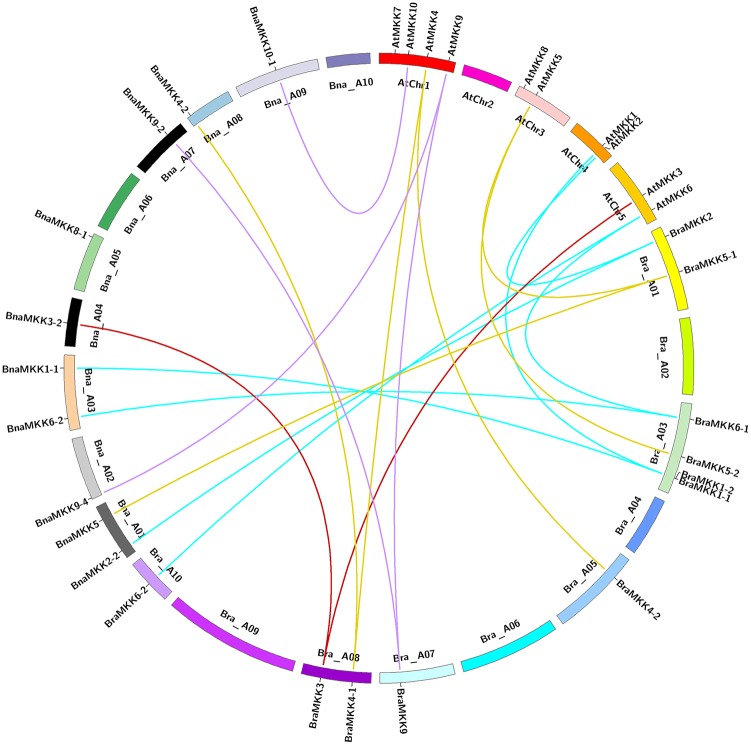
Synteny mapping of *MKK* genes in *B*. *rapa*, *B*. *napus* and *A*. *thaliana* chromosomes. Circle represents each chromosome of *B*. *rapa*, *B*. *napus* and *A*. *thaliana*. Synteny relationships were lined by Circos (http://circos.ca/). Lines with four different colors indicated four groups (A-D) of *MKK* gene family. Genes located on *B*. *napus* A genome are syntenic with genes of *B*. *rapa* and *A*. *thaliana*.

**Fig 7:**
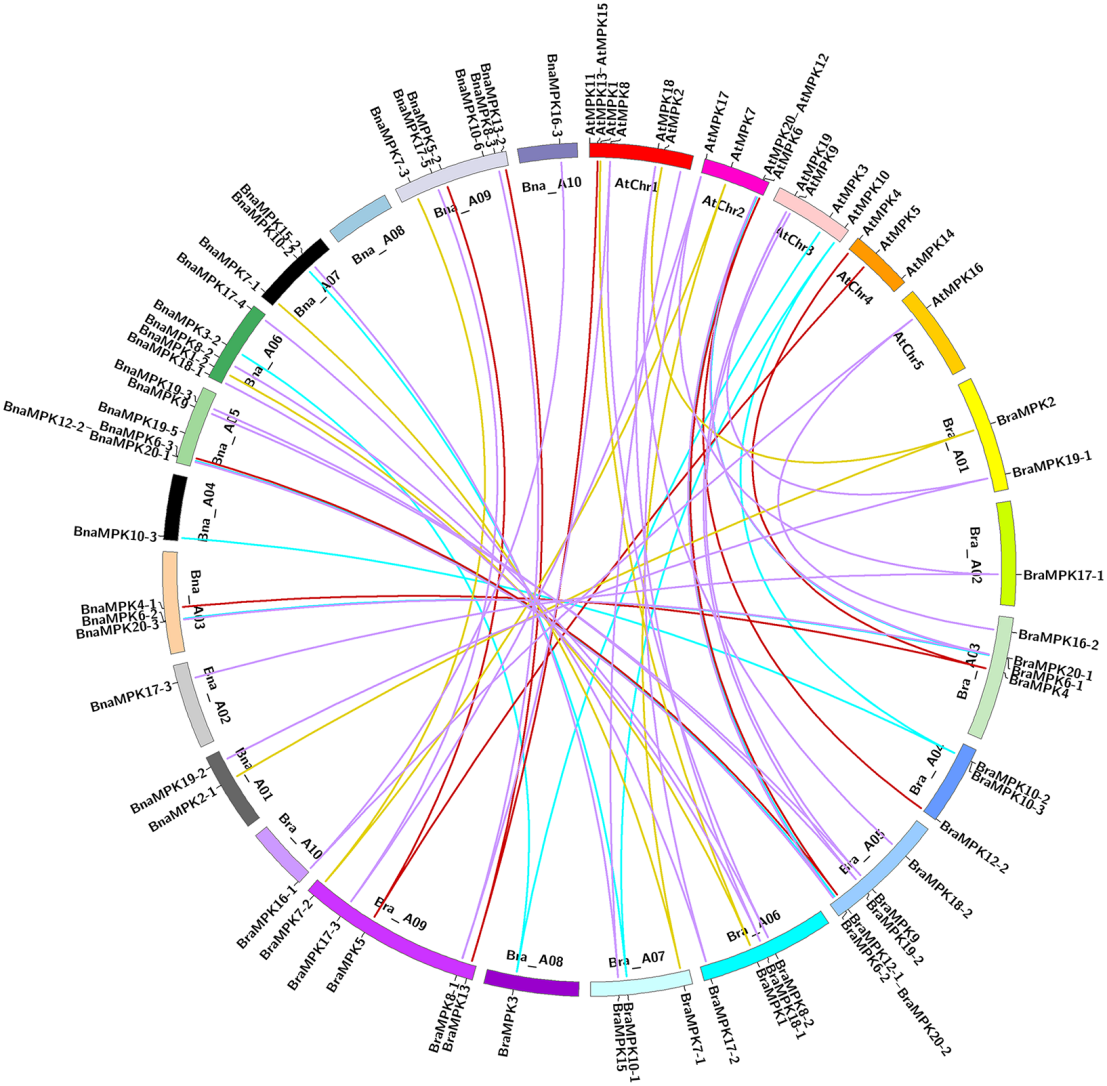
Synteny mapping of *MPK* genes in *B*. *rapa*, *B*. *napus* and *A*. *thaliana* chromosomes. Synteny relationships were lined by Circos (http://circos.ca/). Lines with four different colors indicated four groups (A-D) of *MPK* gene family. Genes located on *B*. *napus* A genome are syntenic with genes of *B*. *rapa* and *A*. *thaliana*.

To comprehensive clarify the mutual relationships of *MKK* and *MPK* genes in the A and C genomes of *Brassica* species, the chromosomal location and orientation of *MKK* and *MPK* genes from *B*. *rapa* (A genome), *B*. *oleracea* (C genome), and *B*. *napus* (A, C genomes) were compared with *A*. *thaliana* (S2 and S3 Tables). Among the 21 *BnaMKK* genes, 10 are located in the A genome and the remaining are located in the C genome (Fig 6 and S4 Fig). Out of the 54 *BnaMPK* genes, 29 and 26 are distributed in the A and C genomes of *B*. *napus*, respectively (Fig 7 and S5 Fig). The synteny map revealed that the *MKK* and *MPK* family genes are conserved, along with the duplication or loss of some genes (S4 and S5 Figs). The orthologous genes of *AtMKK7* were not found in all *Brassica* crops examined. Further, it was found that the *AtMKK8* and *AtMKK10* genes are lost in *B*. *rapa* and *B*. *oleracea*. However, the duplicated genes of *AtMKK8* and *AtMKK10* are present in both A and C genomes of *B*. *napus*. This similar phenomenon was also found in the *MPK* gene family. *MPK11* and *MPK14* are absent in both A and C genomes of *Brassica* species. *MPK10* and *MPK12* were not detected in *B*. *oleracea*, but were found in both *B*. *napus* and *B*. *rapa* genome.

### Expression profiles of *BraMPK* and *BraMKK* genes in different tissues of *B*. *rapa*

**Fig 8:**
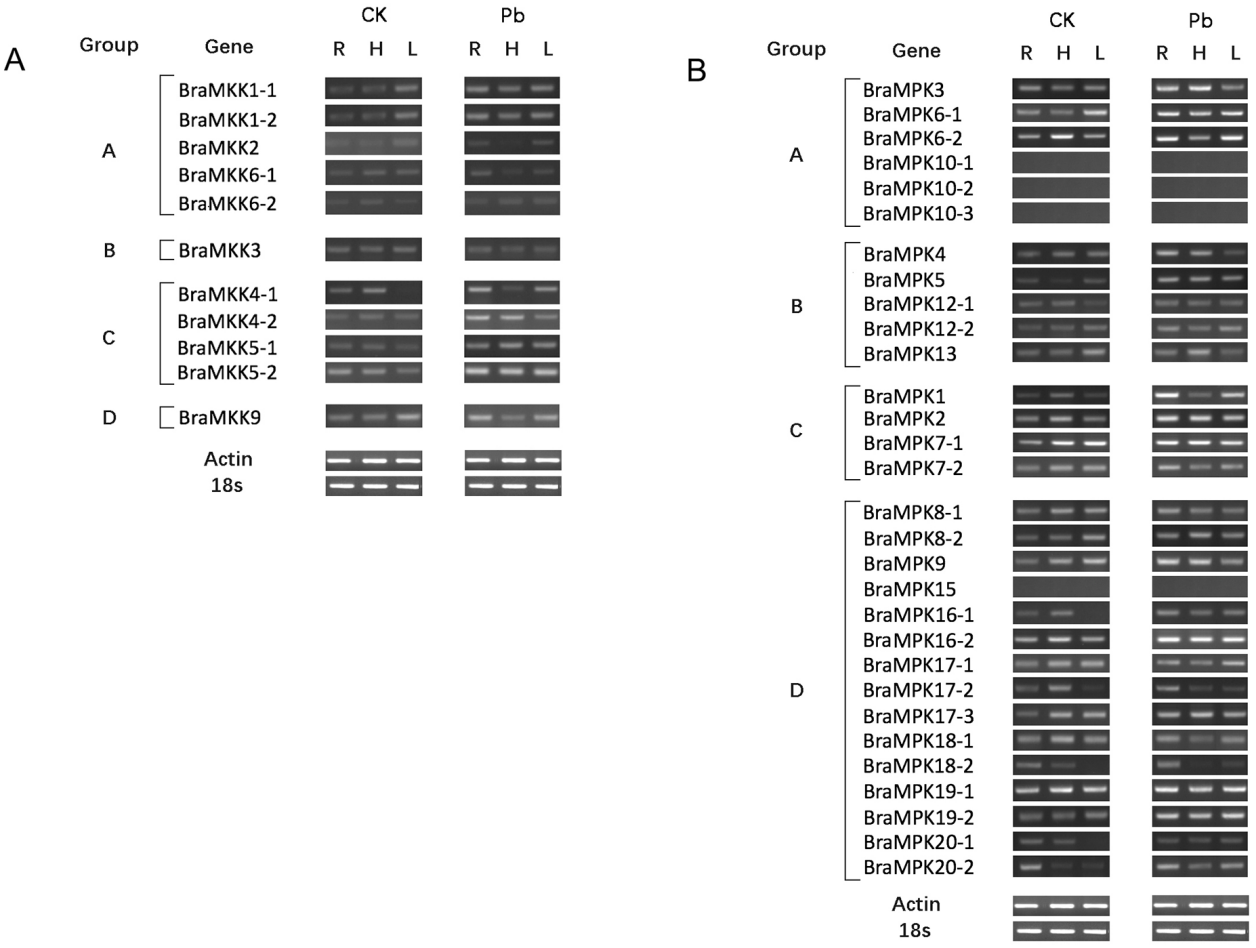
Expression patterns of *MKK* and *MPK* genes in different tissues of Chinese cabbage. Semi-quantitative RT-PCR was used for analyzing tissue specific expressions of *MKK* **(A)** and *MPK* **(B)** genes in *B*. *rapa* (CS NIL). *BraMKKs* and *BraMPKs* are grouped to the phylogenetic classification (A-D). *Actin* and *18s rRNA* were used as internal control. CK: The cDNA pools of control samples. Pb: The cDNA pools of treated samples with *P*. *brassicae*. R: Root, H: hypocotyl, L: Leaf.

To elucidate the expression pattern of *BraMKK* and *BraMPK* genes in natural condition and identify valuable *P*. *brassicae*-triggered *MKK* and *MPK* genes, a semi-quantitative RT-PCR was performed (Fig 8). It was found that 10 *BraMKK* genes were expressed in the roots, hypocotyls, and leaves of *B*. *rapa*. *BraMKK4-1* was not detected in the leaves. The expression levels of *BraMKK* genes in groups A and B were weaker than those of the *BraMKK* genes in groups C and D in all tissues. The expression of *BraMKKs* showed no difference between different tissues. After inoculation with *P*. *brassicae*, four genes (*BraMKK4-2*, *5–1*, *5–2*, *9*) were up-regulated in roots and hypocotyls. *BraMKK4-1* was up-regulated and induced in roots and leaves, respectively, but was down-regulated in hypocotyls. These five *BraMKK* genes showing up-regulation in roots were selected for further qRT-PCR analysis.

Out of the 30 *BraMPKs*, one gene was ubiquitously expressed in the roots, hypocotyls, and leaves of *B*. *rapa*. Three genes (*BraMPK16-1*, *18–2*, *20–1*) were not expressed in the leaves, but were induced after *P*. *brassicae* infection. Four genes (*BraMPK10-1*, *10–2*, *10–3*, *15*) were undetectable in any of the tissues tested, even after treatment with *P*. *brassicae*. The pair-wise genes in groups A and B showed similar expression patterns, while different expression patterns were observed in groups C and D. After *P*. *brassicae* infection, 16 *BraMPK* genes showed up-regulation only in roots, and were used for further expression survey with the samples collected at different time points. Among the 16 up-regulated genes, five genes (*BraMPK2*, *5*, *6–1*, *16–2*, and *19–2*) were up-regulated in all tissues, five (*BraMPK3*, -4, -7-2, -9, and 17–3) were up-regulated in roots and hypocotyls, *BraMPK1* and *BraMPK6-2* showed up-regulation in roots and leaves. *BraMPK7-1*, -*16-1*, -*17-2*, and -*19-1* were specifically up-regulated in roots. Additionally, *BraMPK13* and *BraMPK20-2* were up-regulated in hypocotyls, but four genes (*BraMPK6-2*, *17–2*, *18–1*, *18–2*) were down-regulated. Three genes were up-regulated (*BraMPK12-1*, -*12-2*, and -*20-2*) and three genes (*BraMPK4*, *-9*, *-13*) were down-regulated in leaves.

### Expression profiles of *BraMAPK* and *BraMKK* genes after *P*. *brassicae* infection

To determine the possible biological effects of *BraMKK* and *BraMPK* genes after stimulation by *P*. *brassicae*, 5 *BraMKK* and 16 *BraMPK* genes showing up-regulation in the roots upon *P*. *brassicae* infection were selected for further expression analysis in the roots of CR and CS NILs at the early-infection stages by real-time PCR. Compared to the control, the majority of *BraMKK* and *BraMPK* genes were up-regulated after inoculation, even though some differential expression tendencies were observed between the two NILs.

**Fig 9:**
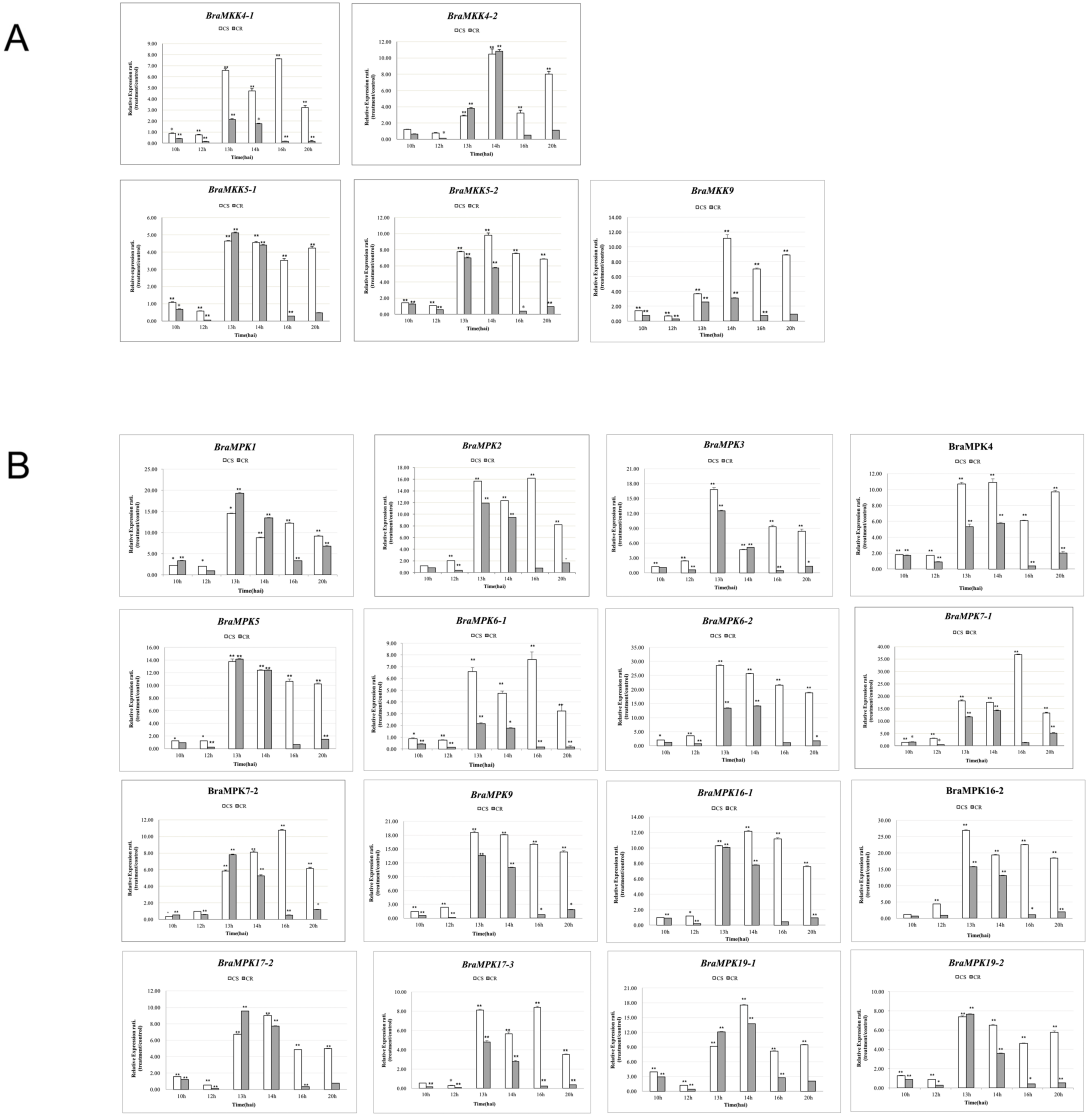
Expression levels of *MKK* (A) and *MPK* (B) genes in roots after treatment with *P*. *brassicae* in both NILs. *Actin* and *18s rRNA* expression levels were used to normalize the data. All data is the mean of 3 biological replicates ± S.E. Significant differences are showed by * (*p*< 0.05) and ** (*p*< 0.01).

After *P*. *brassicae* infection, the expression levels of all *BraMKK* genes were higher in CS NIL than in CR NIL (Fig 9, S5 Table). The *CS BraMKK* genes maintained up-regulation after 12 hai. Although the *CR BraMKK* genes showed high levels of expression after 12 hai, they recovered previous levels after 14 hai. Interestingly, *BraMKK4-1* and *BraMKK9* were strongly induced in the CS BJN3-2 plants, but they showed no difference in the CR BJN3-2 plants. It is worth noting that the pair-wise genes (*BraMKK4-1/4-2* and *BraMKK5-1/5-2*) showed different expression trends in the CR BJN3-2 plants, indicating their functional diversity in response to *P*. *brassicae*.

The *CS BraMPK* genes were strongly induced at 13 hai and maintained the high level of expression even though the folds of expression were fluctuating. After 12 hai, the relative expression levels of the pair-wise genes (*BraMPK17-2/17-3* and *BraMPK19-1/19-2*) were different in each period, while those of the other paralogous pairs were similar. In addition, the *CR BraMPK* genes impetuously reacted at 13 and 14 hai, and restored to normal condition in the later period compared to the *CR BraMKK* genes. The changes in the relative expression quantities of most *BraMPK* genes were higher than those of the *CS BraMKK* genes.

## Discussion

The MAPK cascades play important roles not only in plant growth and development, but also in generating response to biotic and abiotic stresses [31]. Some of the MAPK cascades were found to be involved in plant-virus, -bacteria and -fungus interactions [32]. However, there is no systematic study concerning the effect of MAPK cascades during the interaction between *B*. *rapa* and *P*. *brassicae*. In this study, we identified 11 *BraMKK* and 30 *BrMPK* genes in *B*. *rapa*; these genes were divided into four groups as in *A*. *thaliana*, rice, and other plant species [3, 11, 33, 34]. The use of a pair of near isogenic lines, CR BJN3-2 and CS BJN3-2, allowed us to identify the probable *BraMKK* and *BrMPK* genes participating in *B*. *rapa-P*. *brassicae* interaction.

### Evolution of *BraMPK* and *BraMKK* genes in *B*. *rapa*

Since *Brassica* genomes have undergone whole-genome triplication (WGT) after speciation from *A*. *thaliana* [35, 36], the *B*. *rapa* genome theoretically contains three copies of *AtMPK* and *AtMKK* genes. However, only 11 *BraMKK* and 30 *BraMPK* genes homologous to 10 *MKK* and 20 *MPK* genes in *Arabidopsis* genome were found in the *B*. *rapa* genome. Only *AtMPK10* and *AtMPK17* showed triplication, four *AtMKK* and eight *AtMPK* genes showed duplication, and five genes (*AtMKK7*, *-8*, and *-10*, *AtMPK11*, and *-14*) showed no counterpart in the *B*. *rapa* genome. This suggests that more than half of the *MPK* and *MKK* genes have been lost during the triplication events of *B*. *rapa* genome evolution [37]. Moreover, *MKK8* and *MKK10* were only present in A and C subgenomes of *B*. *napus*, and both *MPK10* and *MPK12* were detected in *B*. *rapa* and *B*. *napus* but not in *B*. *oleracea* genome. This result indicated that these genes were preserved before speciation, but may lost during evolution or artificial selection. The loss of *MPK* and *MKK* genes was also found in other plant species. For example, the homologous genes of *AtMKK7*, *-8* and *AtMPK11*, *-14* were commonly lost in *B*. *rapa*, *B*. *oleracea*, *B*. *napus*, *Piper nigrum* (pepper), *Oryza sativa* (rice), and other species [8, 28, 33, 34, 38–40], indicating that they were not selected during the evolution process. Interestingly, *AtMKK7*, which has no orthologs in *B*. *rapa*, has a function similar to *AtMKK4* in *Arabidopsis* [8]. The retention and duplication of *AtMKK4* in these three species suggests that *MKK4* was probably strongly selected during evolution. In addition, MKK9 was the only gene retained in group D in both *Brassica* and some other species [11, 39]. In previous studies, MKK9 was reported to directly interact with a numbr of MPKs, including BnaMPK1, -2, -5, -9, -19, and -20 in *B*. *napus* [28] and AtMPK5, -10, -17, and -20 in *Arabidopsis* [41, 42], suggesting a possible functional divergence of MKK9 in Brassica species. Our further results suggest that BraMKK9 might be a key member of the MAPK cascade in *B*. *rapa-P*. *brassicae* interaction since its expression was strongly induced after *P*. *brassica* infection in a clubroot-susceptible NIL.

Most of the *BraMKK* genes have similar number of introns compared to other plant species [11, 33]. However, *BraMKK3* contains relatively more introns than the *MKK3* genes in other species, such as *Malus domestica* (8), *A*. *thaliana* (7), *Zea mays* (maize) (8–9) and *Capsicum annuum* (7). It is commonly accepted that the genes responding to stresses contain a small number of introns [43]. *AtMKK4* and *AtMKK5*, which respond to biotic and abiotic stresses, have no intron [8]. The *BraMKK4-1*, -*4-2*, -*5-1*, -*5-2*, *and* -*9* genes, which are up-regulated after *P*. *brassicae* treatment, also have no intron, the same as *C*. *annuum* [33] and *B*. *napus* [28]. In addition, *AtMPK3*, *-4*, and *-6* involved in biotic, abiotic, and hormone responses contain relatively few introns[44, 45]. However, the *BraMPK* genes with more introns were also found be induced by *P*. *brassicae* infection.

### Expression profiles of *BraMPK* and *BraMKK* genes in different tissues and in response to *P*. *brassicae*

In this study, we found that the majority of *BraMKK* and *BraMPK* genes are expressed in the leaves, hypocotyls, and roots of Chinese cabbage, suggesting that they are involved in plant growth and development as in other plant species [41, 46]. The expression of *BraMPK10*-*1*/-*2*/-*3*, and *BraMPK15* were undetectable under normal conditions, even after *P*. *brassica* infection, indicating that these genes functioned as pseudogenes. The most expressed *BraMKK* and *BraMPK* genes showed no differential expression pattern between different organs, although a few genes showed relatively low or no expression in leaves. The low-level expression of *MKK* and *MPK* genes was also detected in the leaves of other plant species [33, 34]. The *MKK* genes in group A showed relatively low expression in the leaves, hypocotyls, and roots of *B*. *rapa*. However, the precise biological significance of the uneven expression of these genes remains to be further studied.

The transcriptional levels of MAPK cascades are largely regulated after environmental stimulation, and the genes transcriptionally modified against the challenge of stress might play important roles in plants’ response to stress [47–49]. In general, the components of MAPK cascades are immediately activated in response to the biotic and abiotic stimuli before 12 hai [11, 28, 33]. In this study, half of the *BraMKK* and *BraMPK* genes were constantly induced in the roots of the clubroot-susceptible genotype ‘CS BJN3-2’ at 13 hai. This is due to the fact that the spores of *P*. *brassicae* can penetrate the root hair at 12 hai [30]. The induction of *MKK* and *MPK* genes by the SA treatment and biotic stresses was also found in other plant species [11, 33, 50]. For instance, the *AtMKK4* and *AtMKK9* genes were activated by the SA treatment, which can regulate the responses to biotrophic pathogens [13]. In agreement, their orthologous genes *BraMKK4-1/4-2* and *BraMKK9* were highly induced in the CS BJN3-2 plants, but were not changed or slightly repressed in the CR BJN3-2 plants. The up-regulation of these genes suggested that they act as the positive regulators of the *B*. *rapa*-*P*. *brassicae* interaction. The distinctive expression patterns of the *BraMKK* and *BraMPK* genes in the two NILs might have resulted from the expression of the *CRb* gene. In CR BJN3-2 plants, the R gene could recognize the *P*. *brassicae* effectors and transmit the signals to the downstream components. Thereafter, the signals might modulate the defense hormone synthesis and/or signaling, defense gene activation, cell wall modification, and phytoalexin accumulation to defend against pathogens. Subsequently, the expression levels of *MKK* and *MPK* genes might recover to the normal level because of the activation of downstream reactions. Remarkably, *BraMPK16-2* was strongly induced from 12 hai and constantly expressed at high levels in the CS BJN3-2 plants, suggesting its key role in the *B*. *rapa*-*P*. *brassicae* interaction. However, no information is available to prove that it was a response to pathogens in any other plant species. Thus, further analysis is needed to determine which *BraMKKs* and *BraMPKs* are the direct upstream components of BraMPK16-2.

### Possible modules of MKKs and MPKs involved in *B*. *rapa-P*. *brassicae* interactions

Plants have evolved a delicate and complicated immune system to protect plants against the attack by microbial pathogens [7]. Plant PRR proteins can recognize pathogen/microbe-associated molecular patterns (PAMPs) to trigger pattern-triggered-immunity (PTI) signaling, the first layer of inducible immunity. PTI provides the basal resistance to a wide range of pathogens, including fungi, bacteria, oomycetes, nematodes, and viruses [7, 51, 52]. After the recognition of a PAMP by PRR, a complex defense network is activated at the early cellular stages, such as the production of reactive oxygen species (ROS), MAPK cascades phosphorylation, and hormone signaling events [53]. Finally, transcription factors, such as WRKY [54, 55], are phosphorylated to promote the expression of stress resistance-related genes [56].

Numerous studies have functionally characterized several important MAPK cascades involved in plant-microbe interactions. It is well known that the modules of MEKK1-MKK4/5-MPK3/6 and MEKK1-MKK1/MKK2-MPK4 and their downstream targets WRKY22/29 regulate plant immune responses against *Pseudomonas syringa*e in *Arabidopsis* [13, 15]. The *Arabidopsis* mutant lines of *mkk1mkk2* and *mpk4* showed enhanced resistance to biotrophic pathogens and increased susceptibility to necrotrophic fungi [17, 57]. In this study, we found that three pair-wise genes (*BraMKK4-1*/*4-2*, *BraMKK5-1*/*5-2*, and *BraMPK6-1/6-2*) and *BraMPKs*, *MPK3* and *MPK4*, were strongly and continuously activated in the roots of the CS BJN3-2 plants, but *BraMKK4-1* and *BraMKK5-2* showed weak up-regulation in the CR BJN3-2 plants. Furthermore, the up-regulation of WRKY22/29 was found in our previous study [30]. Therefore, it could be speculated that the elicitors secreted by *P*. *brassicae*, a biotrophic pathogen, can induce the activation of *BraMKK4-1/4-2* and B*raMKK5-1/5-2*, which in turn activates *BraMPK3*, *-4*, and *-6*, leading to gall formation in the roots.

The cascade of LeMAPKKKα-LeMKK2-LeMPK1/LeMPK2/LeMPK3 has been identified in tomato-*Hyaloperonospora parasitica Noco2* interaction [58]. The expression level of *BraMKK2* was not changed after *P*. *brassicae* infection, indicating that this cascade was not related to the *B*. *rapa*-*P*. *brassicae* interaction. Except for the above-mentioned *BraMPK* genes that are involved in the well-known plant-microbe interaction, several other *BraMPKs* were found to respond to *P*. *brassicae*. In *B*. *napus*, BnaMKK9-BnaMPK1, -2, -5, -9, -19, -20, and BnaMKK9-BnaMPK5, -9, -19, -20 were associated with response to *P*. *brassicae*. Furthermore, the signaling module of MKK9-MPK19-WRKY20 was reported to be responsible to *Sclerotinia sclerotiorum* in *B*. *napus* [28]. The *BraMKK9* and *BraMPK19-1/19-2* genes were also significantly induced in the *P*. *brassicae*-infected CS BJN3-2. The *AtMKK9* gene was reported to induce the biosynthesis of camalexin, which is related to the defense response to *P*. *brassicae* in *Arabidopsis* [59]. In the modules of MKK9-MPK1/2-WRKY53, MKK9-MPK5, and MKK9-MPK9/19/20, the transcripts of *BraMKK9*, *BraMPK1*, *-2*, *-5*, *-9*, *-19*, and -*20-1* were increased in *B*. *rapa* after *P*. *brassicae* infection. The induction of *CaMPK3*, -*4-1*, -*6-1*, -*6-2*, -*7*, and -*9-1* was revealed in the interaction between *C*. *annuum* and *Ralstonia solanacearum*. However, in the MEKK1-MKK1/2-MPK4 module found in the *Arabidopsis-Pseudomonas syringae* interaction [60], only *BraMPK4-1/4-2* showed up-regulation. The results obtained in this study indicated that the MEKK1-MKK4/5-MPK3/6-WRKY22/29- and MKK9- mediated modules might be involved in the defense response to *P*. *brassicae* in *B*. *rapa*. Further studies are needed to unveil the precise MAPK cascades involved in *B*. *rapa-P*. *brassicae* interactions by using yeast two hybridization assays.

## Conclusion

So far, some components of the MAPK cascades have been studied in some plant species, such as *Arabidopsis*, *B*. *napus*, *B*. *rapa*, *O*. *sativa*, and others, while no systematic study has been carried out on the interaction of *B*. *rapa* with *P*. *brassicae*, the causal agent of clubroot disease in Brassicas. In this study, we identified a total of 11 *BraMKK* and 30 *BraMPK* genes, which were unevenly distributed in 6 and 10 chromosomes, respectively. Both BraMKKs and BraMPKs were classified into 4 clades, which were identical to the *Arabidopsis* genes; however, they showed gene duplication and losses. The gene expression analysis revealed that most of the genes were expressed in different tissues. Further analysis indicated that half of the *BraMPK* and *BraMKK* genes were expressed in response to *P*. *brassicae* infection, and their expression patterns were different between the clubroot-resistant and -susceptible genotypes. We speculated that the possible MAPK cascades involved in *P*. *brassicae*-*B*. *rapa* interaction were BraMKK4/5-BraMPK1/3/6/7/19 and/ or BraMKK9-BraMPK1/2/5/9/19. The results obtained in this study will provide information to uncover the biological roles of MAPK cascades involved in *B*. *rapa*-*P*. *brassicae* interaction.

## Supporting information

S1 Fig Multiple amino acid sequences alignments of BraMKKs. Alignment is performed using Clustal Omega and marked by using BoxShade. Duplicate and similar amino acids are shaded in black and grey, respectively. Amino acids in red box (VGTxxYMSPER) are typical motif of MKKs.
(JPG)

S2 Fig Multiple amino acid sequences alignments of BraMPKs. Alignment is performed using Clustal Omega and marked by using BoxShade. Duplicate and similar amino acids are shaded in black and grey, respectively. Amino acids in red box (T(E/D)YVxTRWYRAPE(L/V)) are typical motif of MPKs.
(JPG)

S3 Fig WebLogo plots of consensus motifs in each BraMKKs (A) and BraMPKs (B). (JPG)

S4 Fig Synteny mapping of *MKK* genes in *B*. *oleracea*, *B*. *napus* and *A*. *thaliana* chromosomes. Synteny relationships were lined by Circos. Lines with four different colors indicate four groups (A-D) of *MKK* gene family. Genes located on *B*. *napus* C genome are syntenic with genes of *B*. *oleracea* and *A*. *thaliana*.
(JPG)

S5 Fig Synteny mapping of *MPK* genes in *B*. *oleracea*, *B*. *napus* and *A*. *thaliana* chromosomes. Synteny relationships were lined by Circos. Lines with four different colors indicate four groups (A-D) of *MPK* gene family. Genes located on *B*. *napus* C genome are syntenic with genes of *B*. *oleracea* and *A*. *thaliana*.
(JPG)

S1 Table MPK genes in *B*. *rapa* genome and their sequence characteristics and physicochemical properties. (XLSX)

S2 Table Chromosomal information of MKK gene family from *B*. *rapa*, *B*. *napus* and *B*. *oleracea*. (XLSX)

S3 Table Chromosomal information of MPK gene family from *B*. *rapa*, *B*. *napus* and *B*. *oleracea*. (XLSX)

S4 Table Primer pairs used in semi-quantitative PCR and qRT-PCR. (XLSX)

S5 Table Original data of qRT-PCR. (XLSX)
