# Supplementary figures and images for: Genome-wide identification and role of MKK and MPK gene families in clubroot resistance of Brassica rapa
Source: PLoS One. 2018 Feb 14;13(2):e0191015. doi: 10.1371/journal.pone.0191015 (PMC5812557; doi:10.1371/journal.pone.0191015)

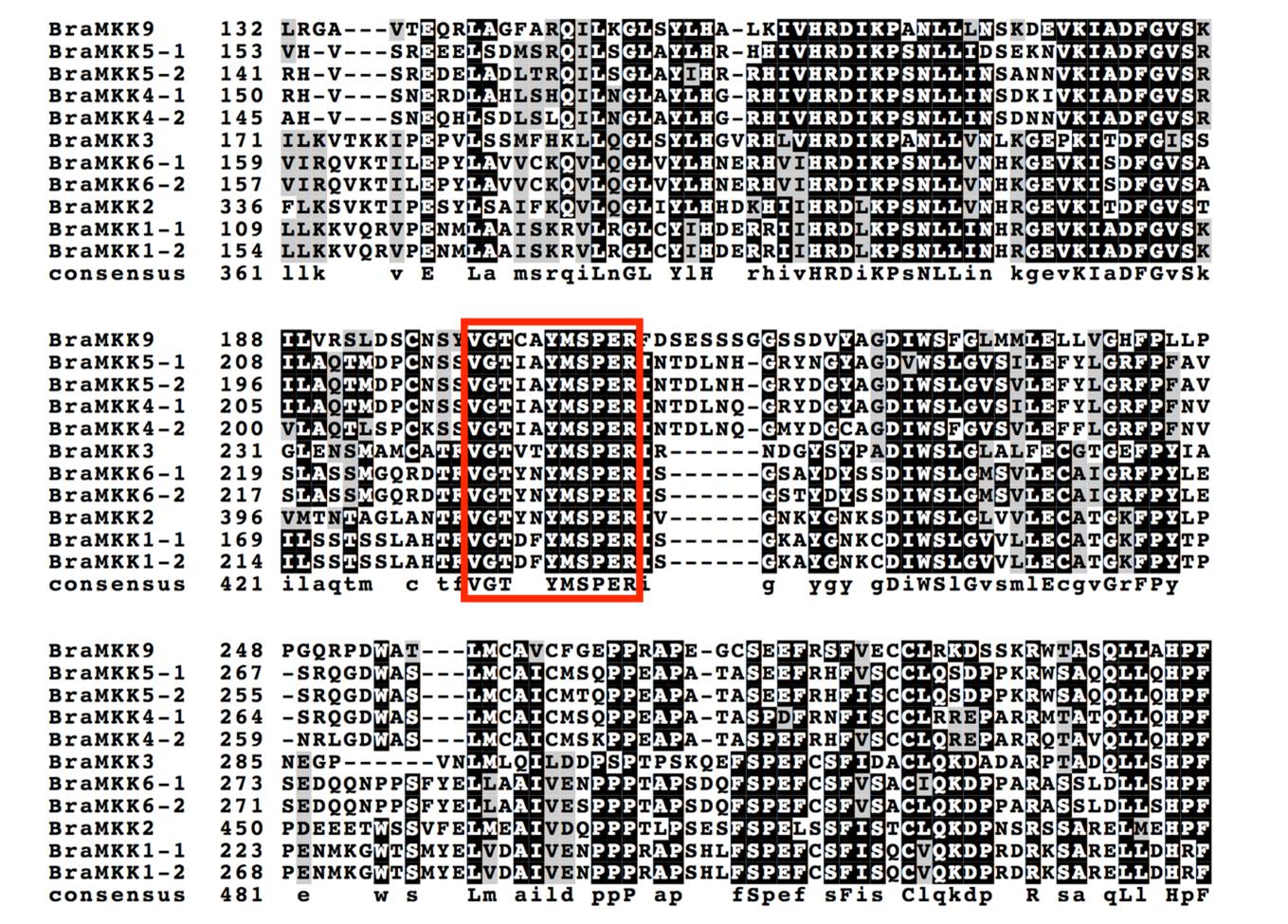

Supplement: S1 Fig — Alignment is performed using Clustal Omega and marked by using BoxShade. Duplicate and similar amino acids are shaded in black and grey, respectively. Amino acids in red box (VGTxxYMSPER) are typical motif of MKKs. (JPG) [file pone.0191015.s001.jpg]

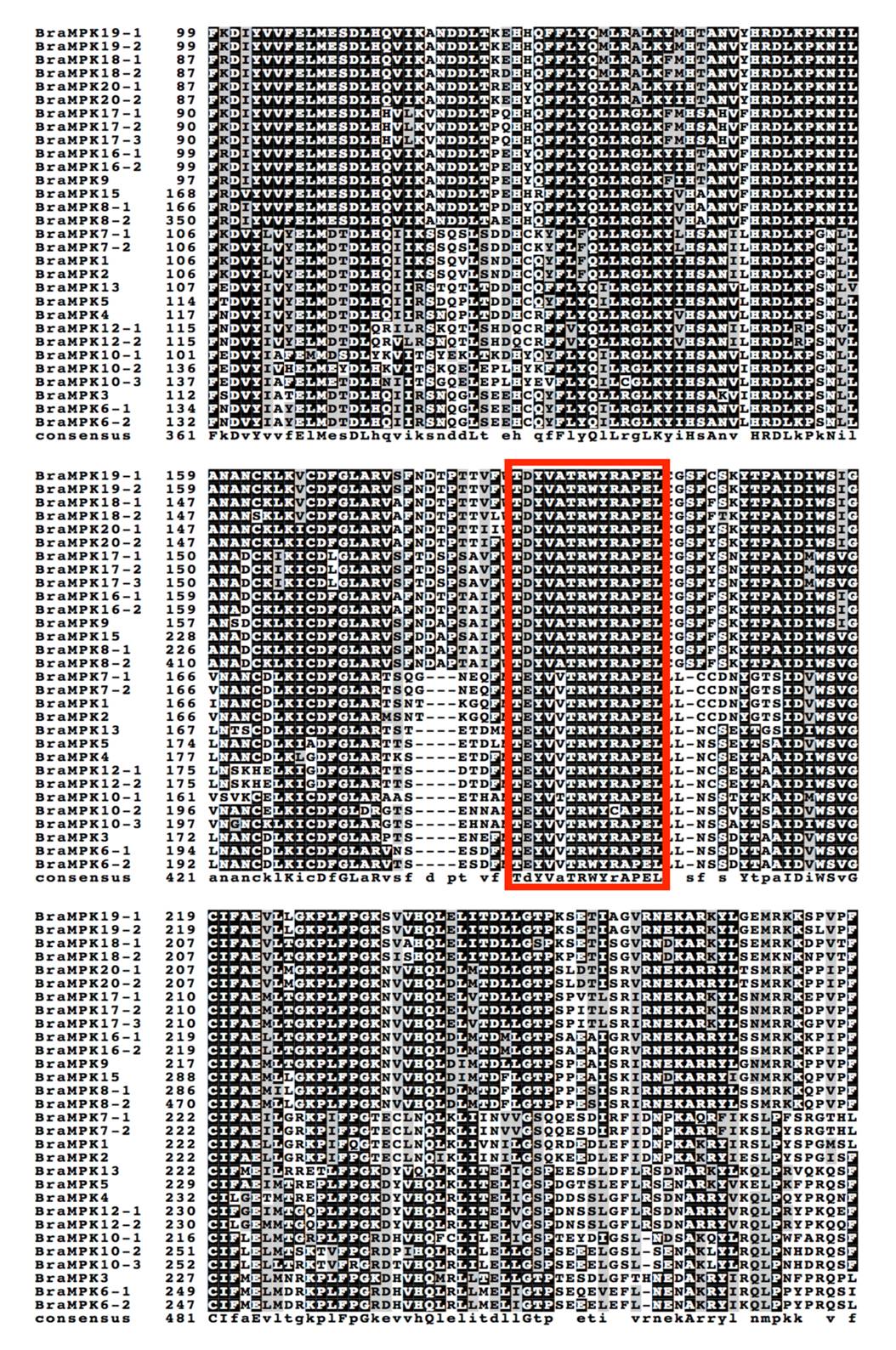

Supplement: S2 Fig — Alignment is performed using Clustal Omega and marked by using BoxShade. Duplicate and similar amino acids are shaded in black and grey, respectively. Amino acids in red box (T(E/D)YVxTRWYRAPE(L/V)) are typical motif of MPKs. (JPG) [file pone.0191015.s002.jpg]

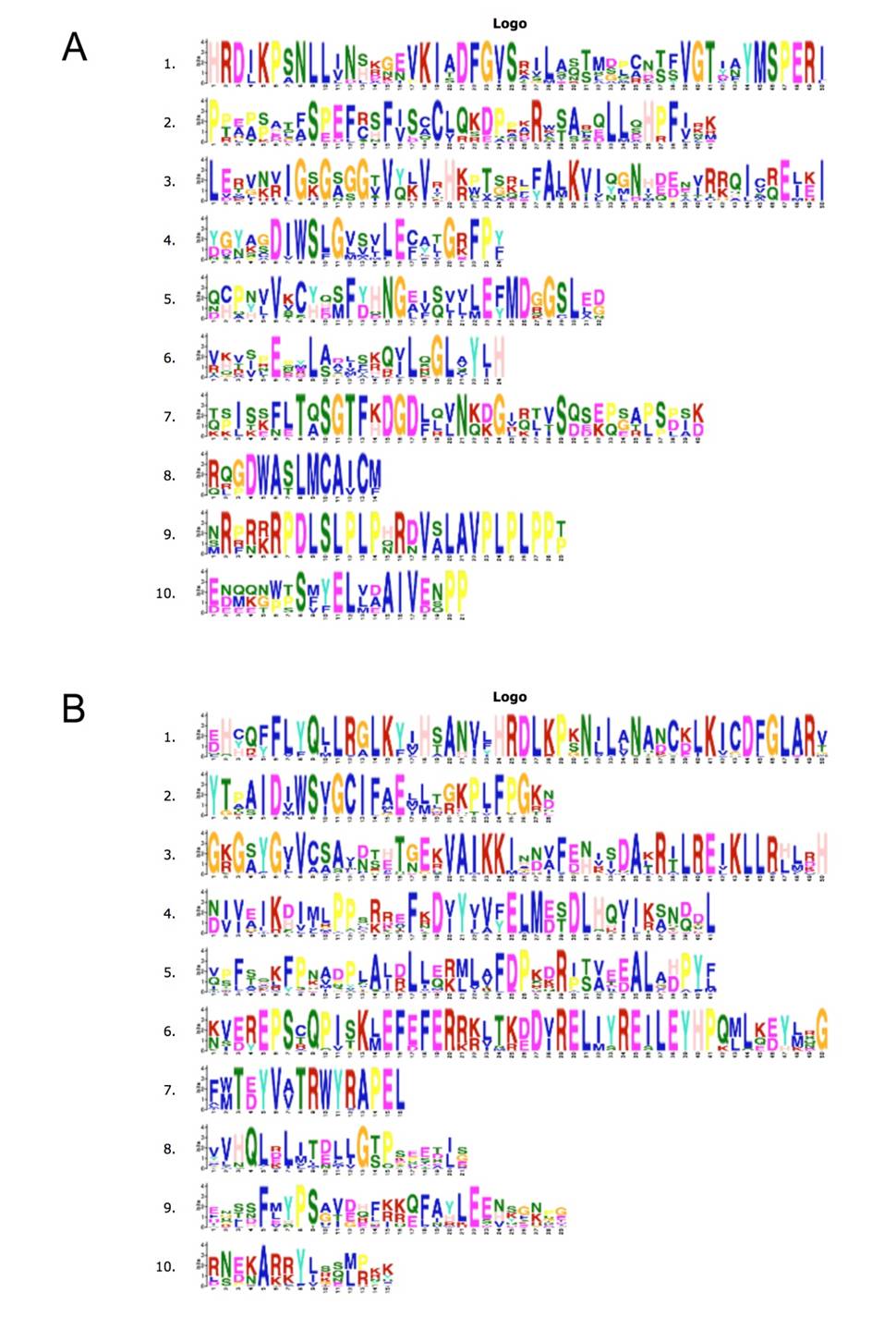

Supplement: S3 Fig — (JPG) [file pone.0191015.s003.jpg]

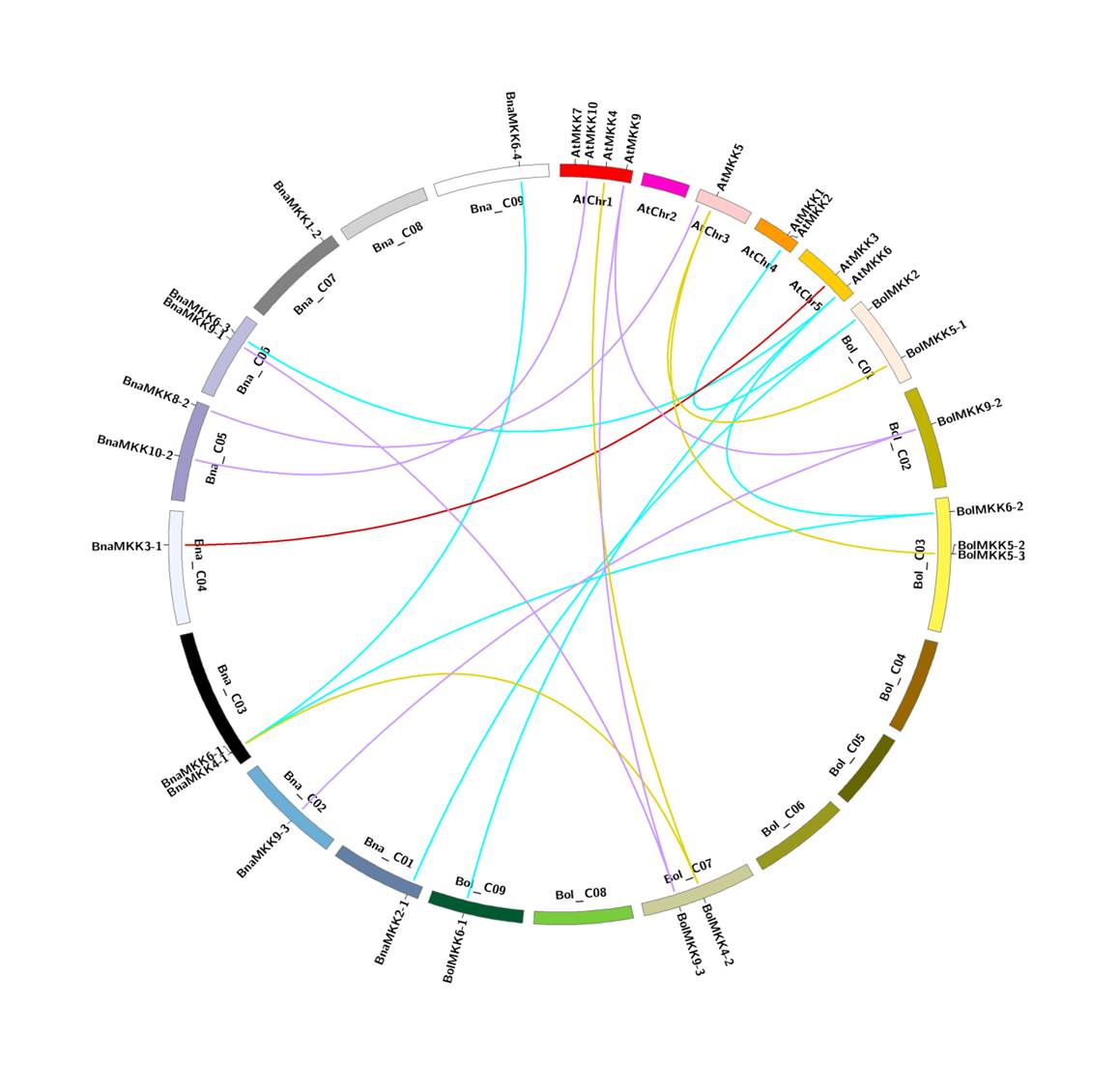

Supplement: S4 Fig — Synteny relationships were lined by Circos. Lines with four different colors indicate four groups (A-D) of MKK gene family. Genes located on B. napus C genome are syntenic with genes of B. oleracea and A. thaliana. (JPG) [file pone.0191015.s004.jpg]

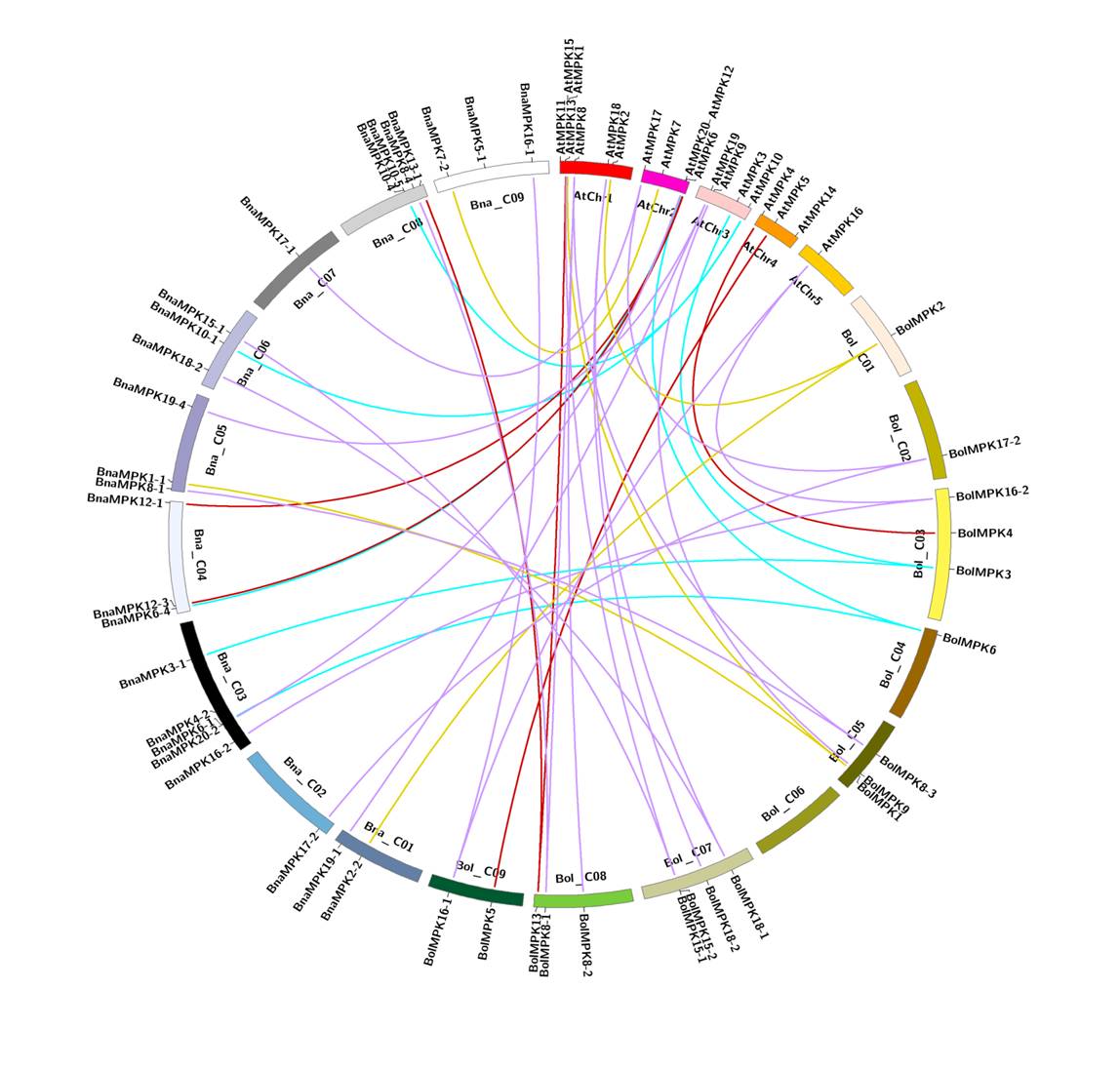

Supplement: S5 Fig — Synteny relationships were lined by Circos. Lines with four different colors indicate four groups (A-D) of MPK gene family. Genes located on B. napus C genome are syntenic with genes of B. oleracea and A. thaliana. (JPG) [file pone.0191015.s005.jpg]
